# The Dog Bite, the Acrometastasis and the Disappearing Hamate

**DOI:** 10.7759/cureus.57246

**Published:** 2024-03-30

**Authors:** Richard Unsworth, John T Hirst, Chelsea L Adam, Jay J Watson, Emma Mulgrew

**Affiliations:** 1 Orthopaedics, Royal Bolton Hospital, Bolton, GBR; 2 Trauma and Orthopaedics, Surrey and Sussex Healthcare NHS Trust, Surrey, GBR

**Keywords:** dog bite wounds, basaloid squamous cell carcinoma, orthopaedics surgery, histological sampling, scintintigraphy, radiology, acrometastasis, hamate

## Abstract

In this case report, we describe a peculiar case of basaloid squamous cell carcinoma of the lung that was first diagnosed from a hamate metastasis. Acrometastases are bony metastases that are located distal to the elbow and knee. They generally become symptomatic only when a primary tumour has been identified. However, in this instance, the patient first sought medical attention following a dog bite to the ulnar side of the wrist, and thus the acrometastasis was diagnosed first, which is uncommon. We discuss the learning points relating to the unusual presentation of this case, classical acrometastatic features and a review of the literature.

## Introduction

Acrometastases are malignant secondary tumours of the skeleton occurring distal to the elbows and knees, most commonly occurring in the hands or feet [1]. Statistically, these rare tumours are most prevalent in the distal phalanx of the dominant hand of smoking males [2]. Approximately 44% of acrometastasis originate from the lung [2]. They are often a pre-terminal event with an average life expectancy of three to six months from diagnosis [2,3].

Studies have demonstrated the canine ability to detect cancer, including those of the lung, in the bodily fluids of humans suffering from cancer [4]. Some authors have argued, however, that local trauma can cause acrometastases via the induction of local inflammation, increased blood flow and release of cytokines promoting the deposition of circulating tumour cells [5]. Therefore, an insult such as a dog bite might promote metastatic deposition at the injury site.

The purpose of this report is to describe the metastasis of a basaloid squamous cell carcinoma of the lung to an unusual location, the hamate, which presented following a dog bite. We aim to impress upon the reader the difficulties of making an early diagnosis of acrometastases and the lessons learnt in its initial management.

## Case presentation

A 54-year-old male smoker presented to emergency services with localised tenderness and swelling about the ulnar border of his left wrist. The patient attributed his symptoms to an unprovoked dog bite four weeks previously. Initial investigations revealed raised inflammatory markers and a ‘normal’ wrist X-ray. Antimicrobial therapy was initiated to treat what was deemed a localised soft tissue infection, and his symptoms initially improved. However, upon cessation of the antibiotics, the symptoms recurred, and a further two-week antibiotic course was prescribed in the community.

**Figure 1:**
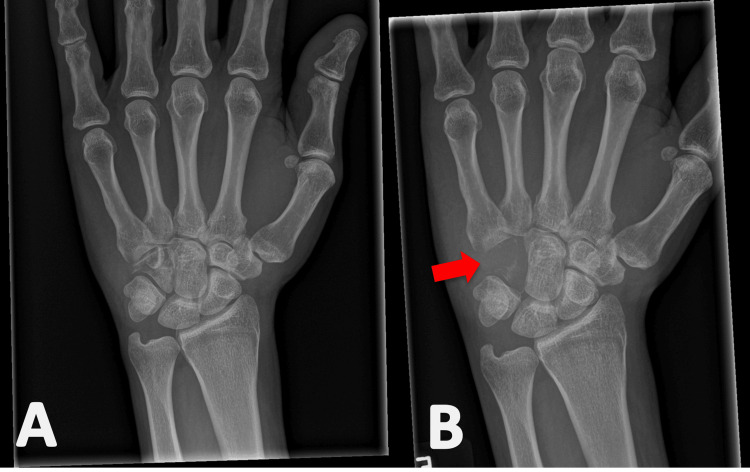
Left wrist posteroanterior radiograph from first presentation to the emergency department showing a normal wrist (A) and eight weeks following the onset of symptoms (B). Profound lytic destruction of the hamate is shown by the arrow.

**Figure 2:**
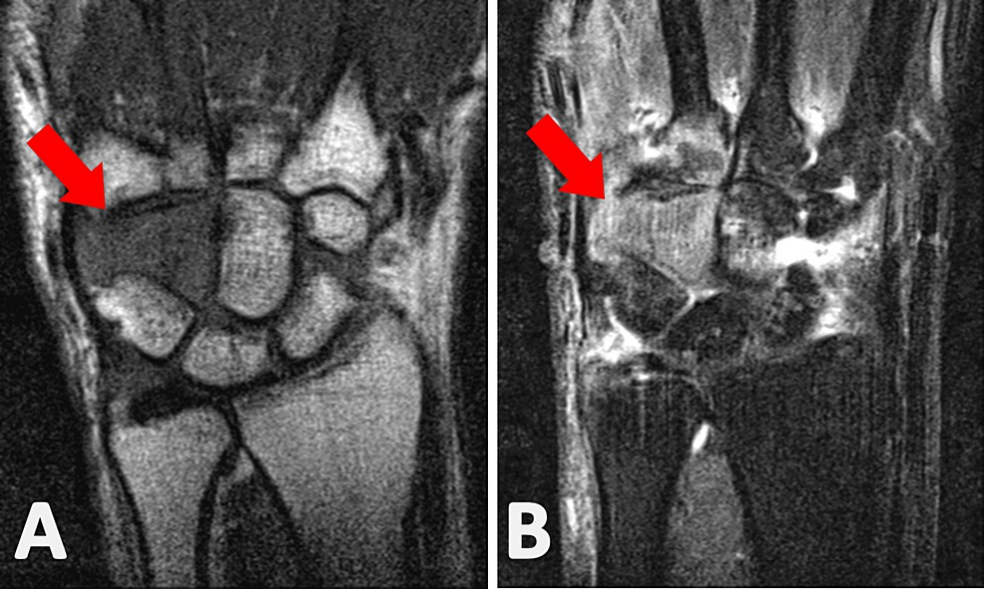
T1 (image A) and T2 (image B) weighted coronal MRI scans of the left wrist, eight weeks from symptom onset. The abnormal signal around the hamate bone area, shown by the arrows, was reported as an inflammatory mass.

Eight weeks following the dog bite, the patient again presented to emergency services with intermittent right-sided pleuritic chest pain. Thoracic examination revealed only minimal tenderness over the right pectoral region. Incidentally, the ongoing wrist pain with fluctuant swelling over the dorsal aspect of his wrist was concurrently noted despite several weeks of antimicrobial therapy. A wrist X-ray revealed almost complete resorption of the hamate bone (Figure 1). An urgent MRI scan was reported as an inflammatory mass measuring 30 x 21 mm centred on the hamate, with extensive marrow abnormality that was not able to be fully appreciated on a plain radiograph (Figure 2).

An open surgical exploration and washout of the wrist was carried out through a dorsal approach. Three millilitres of purulent and gelatinous tissue was drained from the carpal joint. The intraoperative appearance remained consistent with deep infection, and so infection remained the most likely diagnosis. Tissue samples were obtained for microbiological culture and sensitivities along with concurrent histological and cytological samples, in line with departmental policy.

**Figure 3:**
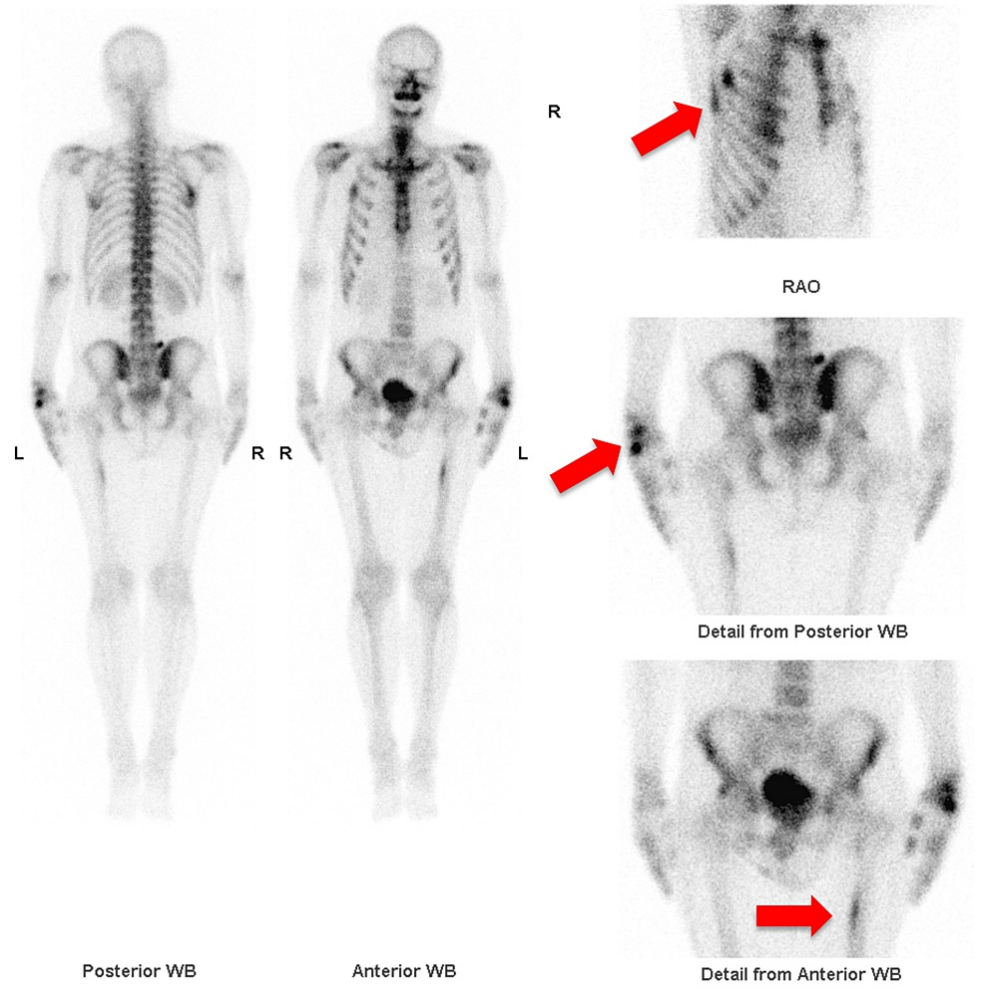
Bone scintigraphy highlights increased metabolic activity in the left wrist, left femur and right ribs (shown by arrows).

**Figure 4:**
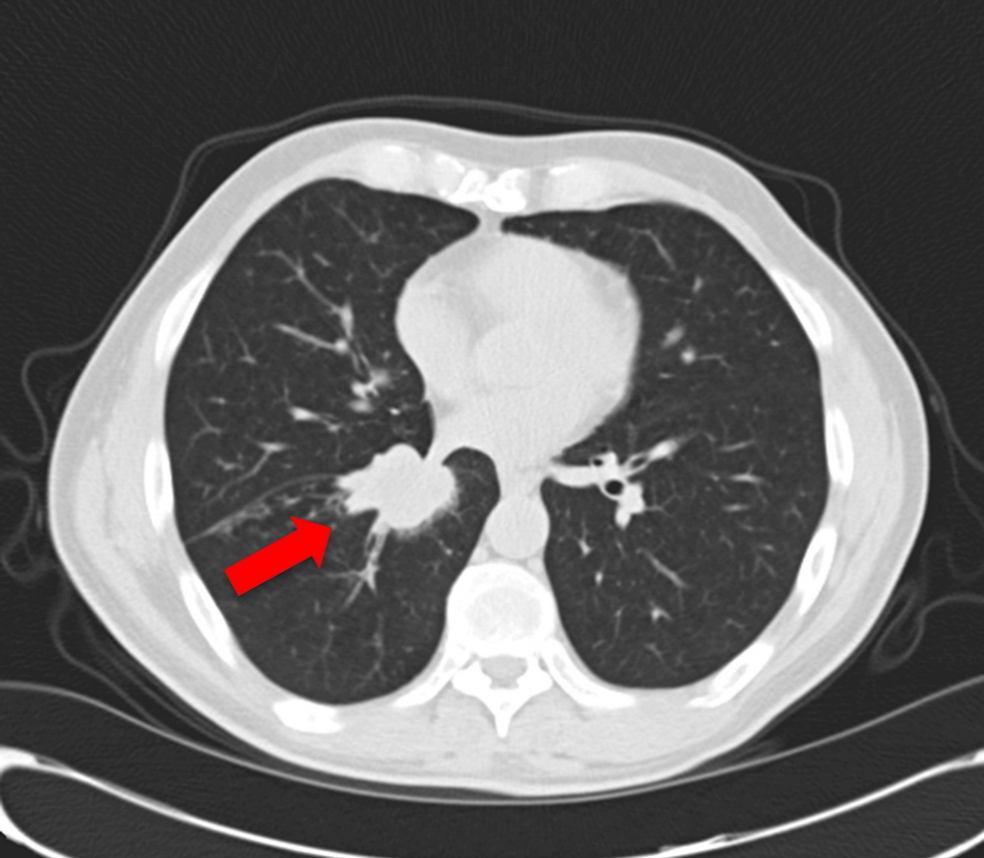
An axial view of the thoracic CT scan reveals a hyperdense mass in the right lung, shown by the arrow.

Broad-spectrum intravenous antibiotics were commenced to cover for possible osteomyelitis because of the intraoperative appearance. However, microbiological cultures did not isolate any organisms. Histology revealed fragments of fibroconnective tissue heavily infiltrated by ‘invasive moderate to focally poorly differentiated basaloid squamous cell carcinoma with focal keratinisation’. Whole body bone scintigraphy (Figure 3) and a CT scan of the thorax, abdomen and pelvis (Figure 4) revealed a right lung primary tumour with additional metastatic deposits within the adrenal glands, femur, ribs and spinal column. The patient was referred to and managed by respiratory specialists following the diagnosis of metastatic lung carcinoma. The patient died less than six months later, as would be expected following this diagnosis.

## Discussion

Diagnosis of acrometastasis is inherently difficult for several reasons. With only one in 1,000-4,000 secondary bony metastases occurring in the hands or feet, their rarity means they are less often considered as a differential diagnosis [1,2]. Additionally, they lack unique identifying features and may mimic osteomyelitis [6], as was initially thought following initial surgery in this case. This case is particularly unusual as 90% of patients have symptoms attributable to their primary tumour before that of their acrometastasis [7]. Acrometastatic spread is predominantly located within the distal phalanges of the dominant hand [1,8] rather than the wrist, further complicating diagnosis in such cases. As such, there is usually a delay in diagnosis and treatment whilst metabolic and inflammatory conditions are usually considered first [9]. However, the primary tumour was, as is typical, a bronchogenic carcinoma of squamous cell origin in a male [2].

In this case, it was the dog bite that drew attention to this region. The dog’s intervention is likely a coincidence, although some authors have argued that local trauma is a contributing factor to acrometastasis. Local trauma induces blood flow, local inflammation and release of cytokines that could promote the deposition of circulating tumour cells [5]. Conversely, authors have also reported on studies where canines have demonstrated the ability to detect cancer in humans by the presence of an ‘odour signature’ in urine, sweat, breath and even blood [4]. It is unclear whether this unprovoked dog attack was caused by, or the cause of, the hamate acrometastasis later found on imaging, or indeed whether it is related at all.

The injury was initially treated as a localized soft tissue infection, which was reasonable given the presenting symptoms and normal wrist X-ray at the time. However, the atypical response to antibiotics in the community should have prompted consideration of further investigations. In modern medicine, there is a shift toward ‘virtual’ management of orthopaedic conditions with self-management, when safe, to avoid unnecessary physical outpatient reviews [10]. Patients need to be advised, however, of the signs or symptoms to be wary of and when to re-present. Specifically, in this case, the recurrence of symptoms, such as pain and swelling despite antibiotic therapy should have prompted a physical review and further investigations. In hindsight, further investigations would not be justified at the initial presentation without systemic symptoms, and thus this case highlights the importance of conducting an opportunistic comprehensive systemic review of all patients. Clinicians need to maintain a wide differential diagnosis when managing these ‘routine’ or ‘straightforward’ cases, especially when patient response to treatment is atypical.

Once the inflammatory mass was identified on MRI, surgical exploration and washout were conducted. In this case, the departmental policy of concurrent histological sampling diagnosed the acrometastasis. This is an important lesson that histopathology samples should be collected with any deep microbiology samples and vice versa. Without this sample, early diagnosis of a lung primary and subsequent targeted treatment may have been delayed.

## Conclusions

This case highlights the rare potential for bony metastases to occur within the hands or wrists as the primary presentation of metastatic cancer. It also highlights the importance of maintaining a broad thought process when managing suspected infections or malignant masses, especially when there is diagnostic uncertainty because of an atypical response to treatment. Samples for both microbiology and histopathology are essential during surgical procedures for accurate and timely diagnosis. The role of the dog is unclear; however, it is interesting to note that its bite on the patient’s wrist led to diagnosis significantly earlier than we would have otherwise expected.
